# Secure LoRa Firmware Update with Adaptive Data Rate Techniques †

**DOI:** 10.3390/s21072384

**Published:** 2021-03-30

**Authors:** Derek Heeger, Maeve Garigan, Eirini Eleni Tsiropoulou, Jim Plusquellic

**Affiliations:** 1 Sandia National Labs, Albuquerque, NM 87123, USA; 2 Department of Electrical and Computer Engineering, University of New Mexico, Albuquerque, NM 87131, USA; eirini@unm.edu (E.E.T.); jplusq@unm.edu (J.P.); 3 Roper Solutions, Inc., Las Cruces, NM 88007, USA; maeve@ropertag.com

**Keywords:** cattle monitoring, LoRa, firmware update

## Abstract

Internet of Things (IoT) devices rely upon remote firmware updates to fix bugs, update embedded algorithms, and make security enhancements. Remote firmware updates are a significant burden to wireless IoT devices that operate using low-power wide-area network (LPWAN) technologies due to slow data rates. One LPWAN technology, Long Range (LoRa), has the ability to increase the data rate at the expense of range and noise immunity. The optimization of communications for maximum speed is known as adaptive data rate (ADR) techniques, which can be applied to accelerate the firmware update process for any LoRa-enabled IoT device. In this paper, we investigate ADR techniques in an application that provides remote monitoring of cattle using small, battery-powered devices that transmit data on cattle location and health using LoRa. In addition to issues related to firmware update speed, there are significant concerns regarding reliability and security when updating firmware on mobile, energy-constrained devices. A malicious actor could attempt to steal the firmware to gain access to embedded algorithms or enable faulty behavior by injecting their own code into the device. A firmware update could be subverted due to cattle moving out of the LPWAN range or the device battery not being sufficiently charged to complete the update process. To address these concerns, we propose a secure and reliable firmware update process using ADR techniques that is applicable to any mobile or energy-constrained LoRa device. The proposed system is simulated and then implemented to evaluate its performance and security properties.

## 1. Introduction

Internet of Things (IoT) devices continue to proliferate across consumer, industrial, and agricultural sectors as advances in mobile computing and networking make intelligent automation and sensing both technically feasible and cost-effective. A primary limitation of wireless IoT devices is energy consumption as they are typically powered by low-capacity batteries. These devices connect to the Internet directly through Wi-Fi or cellular, or through a gateway for radio frequency (RF) communication protocols, such as Zigbee, Long Range (LoRa), and Bluetooth. A class of communication systems designed and optimized for IoT exists known as low-power wide-area network (LPWAN) technologies that includes LoRa wide-area network (LoRaWAN), Sigfox, Narrow Band IoT, and Long Term Evolution Category M1 (LTE-M) [1].

LoRaWAN is a protocol developed by the LoRa Alliance meant to reduce the challenges of IoT device implementation by providing gateways in urban environments that receive data from devices and forward it to the cloud. Its security has been vetted thoroughly and it can communicate over very long distances using minimal power [2,3]. There are three classes of the LoRaWAN protocol which trade off communication flexibility for total energy consumption. LoRaWAN is built on top of LoRa, which is a closed source protocol that uses chirped spread spectrum to enable long range communications while sacrificing data rates. LoRa has many configurable parameters, such as bandwidth, spreading factor (SF), preamble size, and error correcting rates, enabling the device to communicate at different data rates depending on the range required.

**Figure 1:**
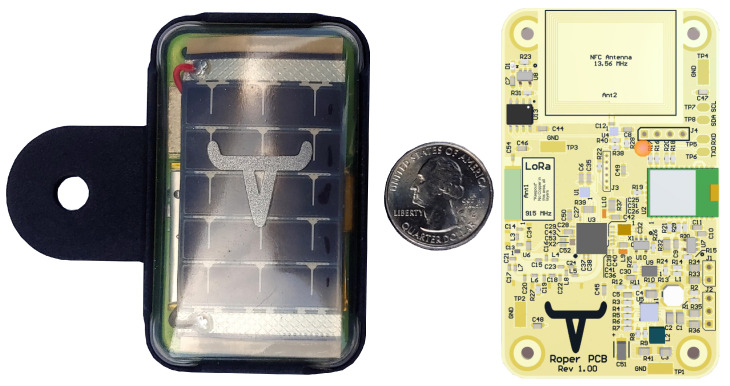
Roper device in housing and the sensor board printed circuit board (PCB).

This research is motivated by the development of a battery-powered LoRa device from Roper Solutions used to track the location and health of free-range cattle, as shown in Figure 1 [4]. This device is comprised of a global positioning system (GPS) module, accelerometer, LoRa communications module, microcontroller, solar panel, and battery. The device periodically collects location and activity data and then transmits it to a custom base station. While this system uses LoRa, it is unable to use LoRaWAN since none of the three classes support the requisite energy efficiency and complex bi-directional communication operations, such as mesh networking and firmware updates. LoRaWAN Class C supports complex communication operations but also requires that devices operate with their receivers always powered on. This requirement represents an unacceptable energy consumption burden because Roper devices are highly size- and weight-constrained and thus use a small, low-capacity battery.

The work presented in this paper focuses on the firmware update process. The time to complete a firmware transfer of a 128 kB image is given in Table 1, presented as a function of the LoRa speading factor (LoRa SF) and various frequency shift keying (FSK) data rates. The 128 kB firmware image requires 2000 data transfers with a 64-byte data packet size. Table 1 shows that this firmware update process takes an unacceptably long period of time at SF = 12, takes a reasonable amount of time at SF = 6, and is fastest when using FSK. An objective of this work is to devise energy efficient methods that achieve high communication data rates with a reasonable bit-error rate using adaptive data rate (ADR) techniques. While our emphasis is on firmware updates, these ADR techniques are applicable to any large data exchange between LoRa-enabled devices. Other examples of large data exchanges include sensor updates from high resolution sources, such as camera images, Lidar, or audio. Bulk data transfers may also occur when a sensor does not have a reliable RF link, so data collected for an extended period of time must be transmitted when the link is reliable.

In addition to the challenges associated with ADR, reliability and security considerations add complexity to the firmware update process. If a firmware update is not completed properly, the device is likely to become inoperable. Since free-ranging cattle can travel long distances, it is quite possible that they will roam outside of the operational communication range of the base station, increasing the risk of an incomplete firmware image transfer. In addition, the device’s low-capacity battery may not be able to provide the power required to complete the firmware update process, resulting in an incomplete transfer. The security of the update process is also a concern since a malicious actor could try to hijack the firmware during the update process, which could allow them to access data stored on the device, including the proprietary embedded algorithms. A malicious actor could also subvert the devices using a Denial of Service (DoS) attack by loading code onto the devices that is not functional. This paper addresses the aforementioned concerns by providing techniques that ensure reliability and security, while using ADR to complete the process as efficiently as possible.

### 1.1. Background

There are numerous research publications and applications employing LoRa and LoRaWAN due to their excellent communications range at a given power consumption. LoRaWAN has three classes of operation (A, B, and C) that allow devices to optimize their performance at the expense of battery power when communicating to a LoRa Gateway. For example, LoRaWAN Class A consumes the least amount of power but can only receive data after it transmits. Class C consumes significant power but allows for advanced operations like mesh networking and is always able to receive data. Applications that benefit from LoRa and LoRaWAN include emergency response communication systems after earthquakes [5], livestock monitoring [6], and intelligent transportation systems [7]. Previous research primarily focuses on key technical issues, such as scalability [8], optimal parameter selection [9], multi-hop capabilities [10,11], security [3], and energy consumption modeling [12,13]. The prior work on LoRa rate adaptation emphasizes challenges with LoRaWAN communication errors in congested RF environments [14,15]. A probing algorithm to improve LoRaWAN efficiency is proposed in [9], and hysteresis is added to the existing LoRaWAN ADR scheme to improve performance in [16]. The tuning parameters and convergence time are analyzed in detail in [17]. Challenges with ADR for mobile devices were covered in [18].

In prior work [19], we address challenges specifically associated with ADR in LoRa ad-hoc networks. Dynamic timeouts and error recovery processes were used to maximize the efficiency, and the ADR expanded into using both LoRa and FSK. We then evaluated the performance of this system using simulations and hardware experiments. The ability to update firmware using LoRaWAN, referred to as Firmware Update Over-The-Air (FUOTA), was published by the LoRa Alliance in [20] and a variant of this capability was implemented securely in a STM32L4 in [21]. Subsequent detailed analysis of the energy efficiency of the LoRa Alliance capability was presented in [22,23]. To the best of our knowledge, no prior work has been published on using LoRa for firmware updates independent of LoRaWAN.

### 1.2. Contributions and Outline

This work expands upon prior ADR research in [19] by applying it to a firmware update process. The research described in this paper makes the following contributions:

1. A method is proposed for energy optimized firmware updates for mobile LoRa devices that is both secure and reliable.
2. The method is designed to work in LoRa ad-hoc networks without LoRaWAN.
3. The method leverages LoRa ADR techniques to minimize energy consumption by reducing the firmware image receive time.
4. A battery consumption approximation technique is provided to quantify the process energy expenditure.
5. A security assessment is performed to evaluate privacy and authenticity.

## 2. Materials and Methods

**Figure 2:**
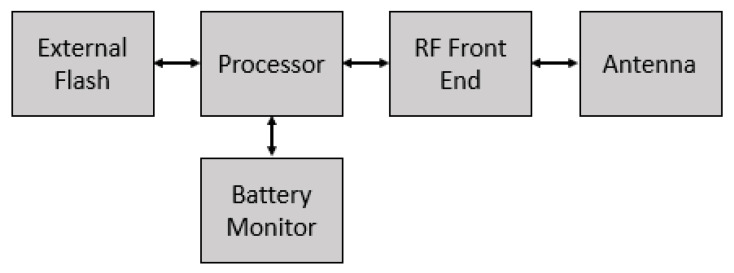
The functional diagram of the hardware required for the firmware update process.

The proposed firmware update process is applicable to any device that uses the LoRa protocol and is equipped with additional memory to store the new code. The memory can be external to the processor or internal if the criteria in Equation (1) is met. Here, $S_{Device}$ designates the total amount of flash memory, $S_{Application}$ represents the flash memory requirements of the application, and $S_{Bootloader}$ represents the requirements for the boot loader. Figure 2 depicts the hardware components associated with the proposed system, and includes components to carry out RF processing, battery monitoring, and an external flash (if required). The battery monitor can vary in complexity but must be capable of approximating the remaining battery capacity, as discussed below in Section 2.3.

(1) $$S_{Device}>=2S_{Application}+S_{Bootloader}.$$

The code resides in flash and is composed of a boot loader and the application. The application code is responsible for implementing the device functionality and must be re-based above the boot loader. The boot loader has the ability to modify and/or erase the application code residing in the flash. The boot loader must also be able to start executing the application code at different addresses depending on where the most recent application update was programmed. The firmware update process is defined as a sequence of five steps outlined below, with most of the functionality residing in the application code. The device will not execute the boot loader code to update the firmware unless it has validated the integrity of the new image and confirmed that there is sufficient battery power to rewrite the flash.

1. Initial Exchange: Establishes status and general configuration information.
2. ADR Rate Optimization: Finds the optimal communication setting with which to exchange the firmware.
3. Battery Approximation: Estimates total energy consumption for completing the update process and terminates the process if the battery capacity is insufficient.
4. Firmware Transfer: Transfers the encrypted firmware image over the RF link.
5. Commit Code to Flash: After confirming that the firmware image is valid, the application hands control over to the boot loader to load the new application code, and then returns control to the new application.

We elaborate on the details of these five steps in the following sections.

### 2.1. Initial Exchange

The primary purpose of the initial exchange is to establish a common understanding of the firmware update status between the base station and receiving device. The base station will initiate the firmware update by sending a short command and the device will respond with the device ID, battery capacity and health status, and current firmware version. The device will also transmit the status of any new firmware that has been received and how much of the data has been transferred into the device. This information is relevant when a partial firmware update has occurred, to avoid re-transferring the same firmware image. The status of the device firmware update could be stored in the base station, but that would prevent the firmware from being loaded by multiple base stations, which is a capability that we want to maintain due to cattle mobility.

**Figure 3:**
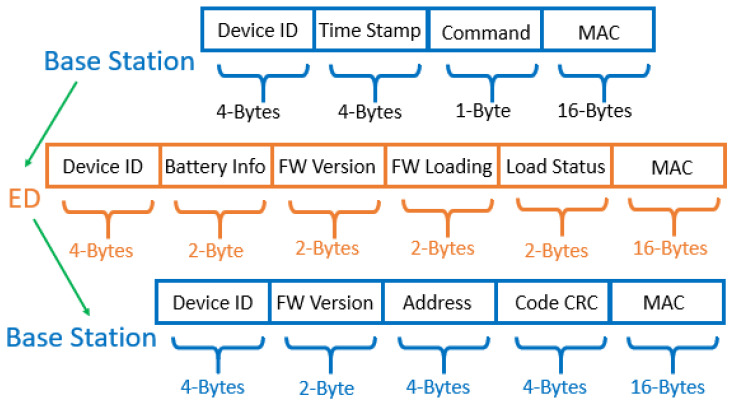
The packet structures for exchanging data during the initialization phase.

If it is determined that a new firmware update is required, the base station will send a new firmware version number, the starting memory address to load the application code, and the cyclic redundancy check (CRC) of the entire firmware image. The starting address is used by the boot loader to instruct it on where to load the code into memory, and the CRC is used to verify that a valid firmware image has been received. If it is determined that an existing firmware update needs to continue, the base station will inform the device and continue from the point of interruption. These communications are signed using Advanced Encryption Standard-Cipher-based Message Authentication Code (AES-CMAC). The details of the packet structures used in the message exchange between the base station and receiving device are given in Figure 3.

### 2.2. ADR Techniques

After defining the firmware update parameters, the next step is to identify the maximum data rate that provides reliable communications. The communications setting has a substantial impact on the total time to transfer data, as shown in Table 1. We leverage the results of prior work on optimizing ADR transfers [19] by applying it to the firmware update process. The parameter search space is limited to the 13 options shown in Table 2. Note that we use all seven LoRa SF in combination with the six FSK bit rates shown in column 3. The extension here over previous ADR work to include the FSK parameters is beneficial because it enables significantly higher data rates when the devices are in close proximity. Moreover, there is no additional hardware required to enable FSK since every LoRa integrated circuit (IC) has FSK capabilities.

This work evaluates two techniques, called incremental search and binary search, which identify the optimal data rate setting using an iterative process, and a third technique that uses the received signal strength indication (RSSI) to intelligently locate the optimal communication setting. The incremental search starts by establishing communications at setting 12, and then the base station commands the device to go to setting 11. The base station listens for the device to acknowledge on setting 11, and then iterates this process decrementing the setting. It continues until the base station fails to receive an acknowledgement because the device signal strength is not sufficient at the base station. Note that the device and base station can only listen on a single setting. This type of setting discovery process adds complexity because when communications fail, the device and base station must revert back to a previous setting to re-establish communications. The base station and the device both have timeouts, as shown in Table 2, that allow them to re-synchronize if communications fail. Note that the magnitude of the timeout values are dependent on the communication setting. This is referred to as the error recovery process which initiates communications at $S_{current}$ and $S_{next}$ in an attempt to re-establish communications with the device.

Binary search uses a methodology similar to incremental search except the next search setting is defined according to Equation (2). Here, $S_{current}$ refers to the setting the devices last successfully communicated on and $S_{highest,failed}$ is the highest setting that failed.

(2) $$\begin{array}{cc} S_{next} & =S_{current}-ceil\left(\frac{S_{current}-S_{highest,failed}}{2}\right) \end{array}.$$

The RSSI optimal search method establishes communications at the worst-case setting (setting 12) and then uses the RSSI level to intelligently select the best $S_{next}$. The RSSI is automatically measured by LoRa ICs when they receive a valid packet so this process does not require special hardware. $S_{next}$ is computed by Equation (3) and is derived from sensitivity specifications of FSK and LoRa (with BW = 125 kHz). RSSI is measured in dBm and should be padded by reducing the measured value by the error tolerance. The hardware measurement error tolerance is 2 to 6 dBm depending on the amount of averaging used. This technique avoids the iterations needed by the other search methods but requires more communications at setting 12 to achieve a reliable RSSI value.

**Figure 4:**
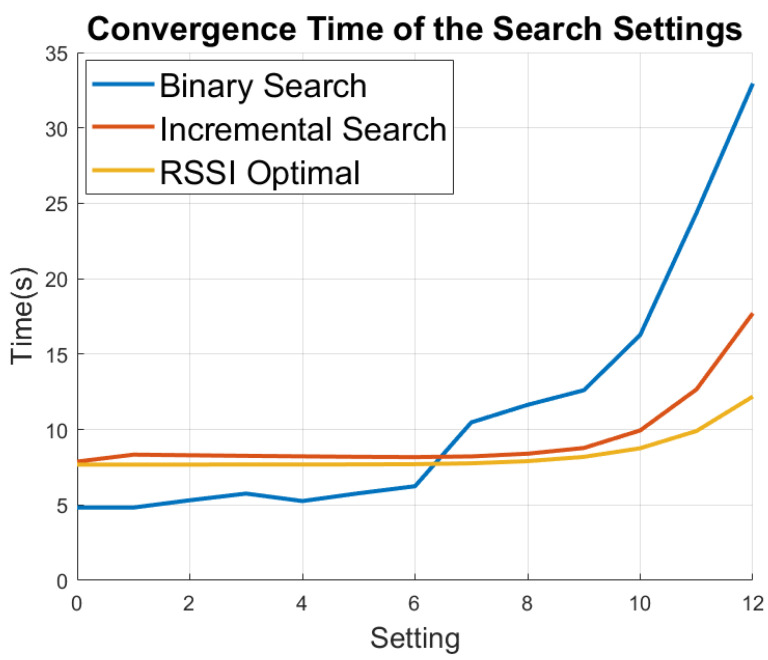
The convergence times of the different ADR search techniques.

Simulations were performed using the search algorithms associated with each technique and the convergence times are shown in Figure 4. The simulation models were configured with the following parameters: (1) bandwidth set to 125kHz, (2) 12-symbol preamble, (3) CRC enabled, (4) implicit header enabled, and (5) error coding rate set to 5/4. The transmitted commands used to find the optimal ADR settings posses a 4-byte payload. The commands utilized to change settings are given as follows: (1) Go to ADR mode, (2) Go to setting, (3) Exit ADR, (4) Ping, and (5) Acknowledgment (Ack), or No Acknowledgement (Nack).

(3) $$\begin{array}{cc} S_{next} & =ceil(-0.0002{\left(RSSI\right)}^{3}-0.0751{\left(RSSI\right)}^{2}-8.7614\left(RSSI\right)-343.76) \end{array}.$$

Binary search is the most efficient technique when converging to lower settings but struggles at the higher settings. This is true because the device must fail more often at the higher settings in order to converge there. Incremental search is more efficient when a higher setting is required because it only fails once, but is slower to converge to a lower setting because it must communicate on every setting. The RSSI optimal search method improves upon incremental search and is best overall at higher settings because the search process is eliminated. However, the additional overhead of obtaining an accurate RSSI value reduces its performance. Further analysis of the ADR method including acknowledgement techniques, starting parameter settings, and the breakdown of transmit and receive time are assessed in detail in [19].

### 2.3. Battery Approximation

After settling on an acceptable communication setting, the device determines whether to initiate the firmware transfer based on the whether the battery has sufficient energy to complete the operation. The total energy consumption is dependent on the size of the firmware image (*i*), the status of an existing firmware update ($i_{s}$), i.e., whether a partial image already exists, and the setting the data is transferred with (*s*). The energy consumption is partitioned into a data transfer portion and a portion required to carry out the application flash write operations as given by Equation (4).

(4) $$\begin{array}{cc} E_{update}\left(i,i_{s},s\right) & =E_{Transfer}\left(i,i_{s},s\right)+E_{AppFlashWrite}\left(i\right) \end{array}.$$

The data transfer can be further subdivided into the energy consumption associated with receiving data, transmitting acknowledgements, performing AES encryption, and writing the flash to store the firmware image, as shown by Equation (5). Note that the flash write operation stores the image on the device but does not commit it to application memory. The electrical current associated with data reception, packet acknowledgement, AES computation and flash write operations is given by $I_{receive}$, $I_{transmit}$, $I_{AES}$, and $I_{extFlashWrite}$, respectively. The transmit and receive time depend on the communication setting but the AES and flash times are fixed. $V_{s}$ is the supply voltage, and η is the efficiency of the switch mode power converter that supplies energy from the battery to the components. For scenarios in which external flash is not being used, $T_{extFlashWrite}$ = 0.

(5) $$\begin{array}{c} E_{Transfer}\left(i,i_{s},s\right)=\frac{\left(i-i_{s}\right)V_{s}}{\eta }(I_{receive}T_{receive}\left(s\right)+I_{transmit}T_{transmit}\left(s\right)+\\ 2I_{AES}T_{AES}+I_{extFlashWrite}T_{extFlashWrite}) \end{array}$$

The energy to write the application flash depends solely on the firmware image size and is broken down into external memory read, AES encryption, and the application flash read and write components, as shown in Equation (6). The external memory read and AES encryption operations occur twice: first to validate the firmware image and second to write the image to flash. For scenarios in which external flash is not being used, $T_{extFlashRead}$ and $T_{AES}$ are set to 0. The specifics of this process are covered in Section 2.5, *Committing Code to Flash*.

(6) $$\begin{array}{c} E_{AppFlashWrite}\left(i\right)=\frac{iV_{s}}{\eta }(2I_{extFlashRead}T_{extFlashRead}+2I_{AES}T_{AES}+\\ I_{flashWrite}T_{flashWrite}+I_{flashRead}T_{flashRead}) \end{array}$$

After the setting and firmware image parameters are defined, the device will use these energy consumption calculations to compute the expected battery capacity after completing the update. This is accomplished using Equation (7) where the capacity *C* is defined as the percent charge left in the battery, similar to what a phone or laptop computer would indicate for battery life. $B_{SOH}$ is the state of health of the battery which begins at 1 and degrades to 0 over time. Rechargeable batteries are generally rated to maintain 80% of full capacity up to 2000 cycles. The energy associated with a fully charged battery ($E_{fullBattery}$) can be calculated using the amp-hour specification for the battery and the average output voltage. The battery capacity estimation techniques used here are based on those presented in [24].

(7) $$\begin{array}{cc} C_{AfterUpdate}\left(i\right) & =C_{Battery}-100\frac{E_{update}}{B_{SOH}E_{FullBattery}} \end{array}.$$

After computing the final capacity value, the device then determines if it can commit to the firmware update process. The exact threshold depends on several factors including the importance of the firmware update, the ability of the system to recharge, the external temperature, the consequence of a discharged battery, and the current capacity of the device. While the cutoff threshold is application-specific, we use a 50% threshold for illustration in this work. Assuming there is adequate energy in the battery, the system will proceed to the firmware transfer step of the update process.

### 2.4. Firmware Transfer

The firmware transfer step involves the base station transferring the firmware image to the device. The packet structure of the transmitted firmware image includes the device ID, the packet number that is being transmitted, 16-bytes of the encrypted firmware image, and a 16-byte MAC to ensure authenticity. The device responds with either an acknowledgement (Ack) or negative acknowledgment (Nack) depending on whether the data arrived reliably and is authenticated via a MAC calculation. The device stores the encrypted data in external memory as it is received. The data is encrypted as a countermeasure to adversarial attacks that attempt to read the image from external memory. If an Ack is received, the base station sends the next packet of the firmware image. If a Nack is received, the base station re-sends the same packet. This process is repeated 8000 times to transfer the entire set of 16-byte encrypted data packets constituting the 128 kB firmware image. If the base station does not receive a response or gets a Nack after three consecutive packet transfer attempts, the base station will re-initiate the ADR to update the optimal communication setting. The base station can also monitor the RSSI of the incoming packets from the device. If there is a significant increase in RSSI, the base station can direct the device to a lower communication setting to speed up the data transfer process.

### 2.5. Committing Code to Flash

After a firmware image is received, the device must first validate it before committing and allowing the image to be executed. Validation begins by reading the image from memory, decrypting it, and then computing its CRC. The CRC computed over the entire image is compared to the CRC value that was sent during the initial exchange. If the two CRCs do not match, the firmware update process is restarted. Additional checks can be performed to increase the confidence that the firmware image is valid, such as checking it against a minimum size threshold and validating the correctness of the initial instructions.

**Figure 5:**
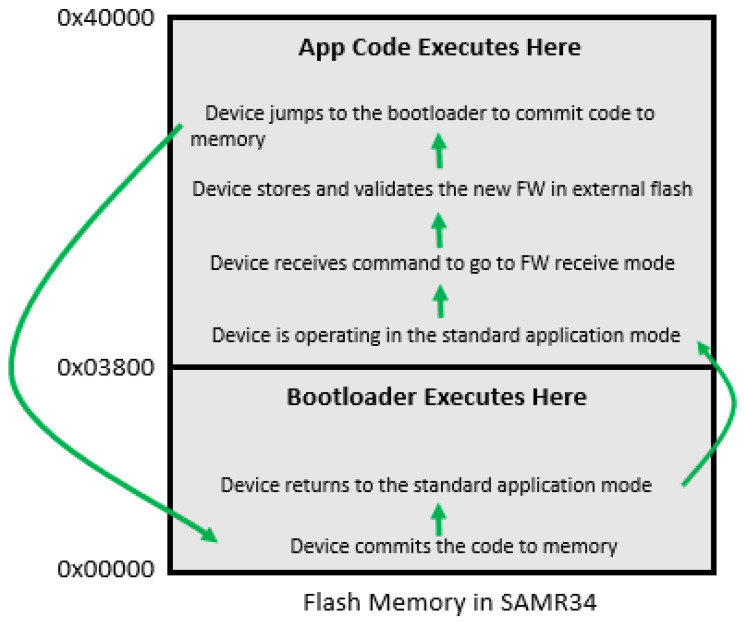
Flash memory breakdown for using external flash.

**Figure 6:**
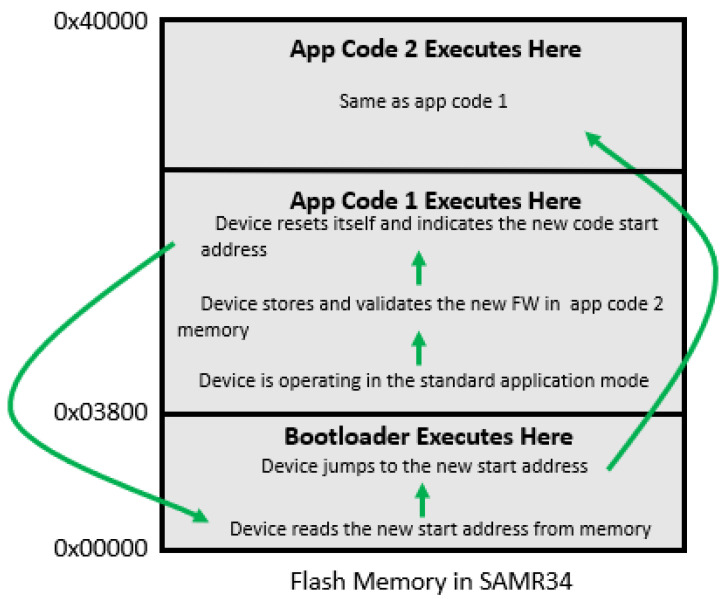
Flash memory breakdown for using internal flash.

Once the image is validated, the system will begin to execute the new application. The process varies significantly depending on whether external or internal memory is used, as seen in Figure 5 and Figure 6, respectively. For the external memory case, the device first confirms that there is sufficient battery to commit the code to flash, and then jumps to the boot loader to begin loading the memory. It reads and decrypts each 64-byte portion of the new firmware update, and then writes the data to flash. After each write, it reads the flash to check that there are no write errors. Upon completion, the processor will reset and jump to the starting address of the application code. In the scenario where external flash is not used, the device bypasses the flash write step since the code already resides in application space. It instead jumps to the starting address (assuming the code is properly re-based) and begins execution. For future firmware updates, it alternates the flash locations between App Code 1 and App Code 2, as indicated in Figure 6.

Before jumping to the new application, all interrupts and processor initialization are disabled and the stack pointer and vector table are re-based. We accomplish this by simply resetting the processor and setting registers in the boot loader to point to the new application code location. This ensures that everything is disabled when the boot loader hands over control to the application. Although it is possible to jump to a new application directly without resetting the processor, doing so mandates that all interrupts and initialization are first disabled which can be tedious.

## 3. Results

The firmware update process described in the prior section was simulated and then implemented on the cattle monitoring sensor. The application flash size met the criteria from Equation (4); therefore, internal flash is used because it is more energy efficient than external flash.

### 3.1. Functional Characterization

**Figure 7:**
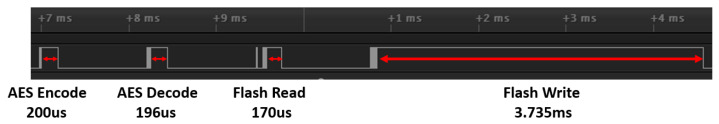
Timing characterization for Advanced Encryption Standard (AES) and flash read and writes.

We first carry out experiments to measure the parameters associated with each component of the transfer process including the AES, flash, and LoRa operations. We utilize a Microchip SAMR34 Xplained evaluation board because it provides a simple interface for measuring current and timing information. Figure 7 shows the measured timing values for the AES encode and decode and flash read and write operations. The flash write is the most expensive operation and cannot be performed without first executing a page erase (the erase time is included in the reported value of 3.735 ms). The LoRa transmit and receive values are setting dependent and were validated in previous work [19]. The parameters used in the following simulations are given in Table 3.

### 3.2. Simulation

**Figure 8:**
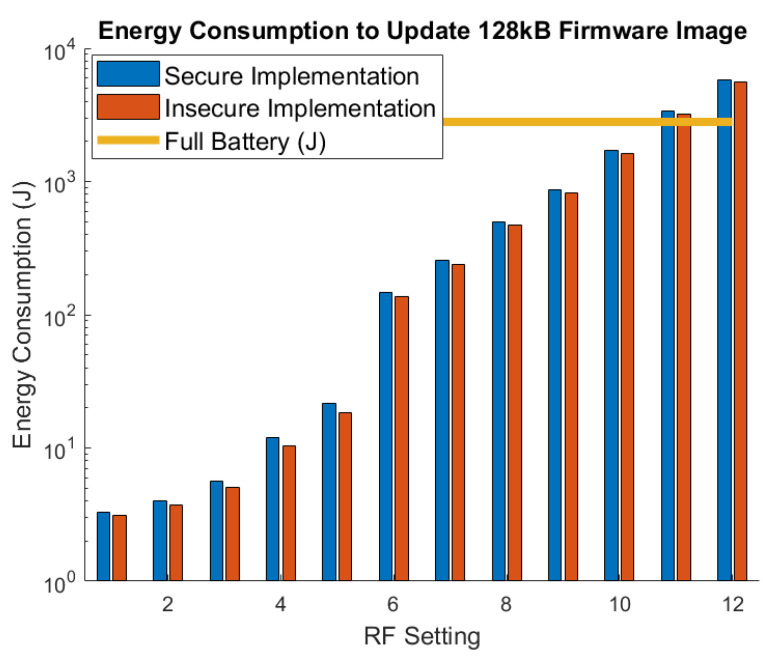
The energy consumption required to update a 128 kB firmware image.

We modeled the energy consumption for a 128 kB firmware image update under both the secure and insecure versions of the proposed system. The performance was modeled using MATLAB because existing LoRa simulator tools were insufficient to quantify total power consumption on the cattle monitoring device. The LoRa transmit and receive characteristics used in the modeling were validated in [19]. The results are shown in Figure 8. The insecure version is similar to the secure version but omits the encryption and MAC operations to authenticate each packet. The orange line indicates the full capacity of a 200 mAh battery which possesses 2.8 kJ. The parameters used in the simulations are given as follows: (1) LoRa Bandwidth = 62.5 kHz, (2) 12-symbol preamble, (3) CRC enabled, (4) implicit header disabled, (5) error coding rate = 5/4, (6) η = 90%, (7) $V_{s}$ = 2.8 V, (8) $I_{transmit}$ = 100 mA, and (9) $I_{receive}$ = 10 mA. Note that our model here excludes the ADR operations, which were covered previously in [19] and the initial exchange because its contribution is negligible.

**Figure 9:**
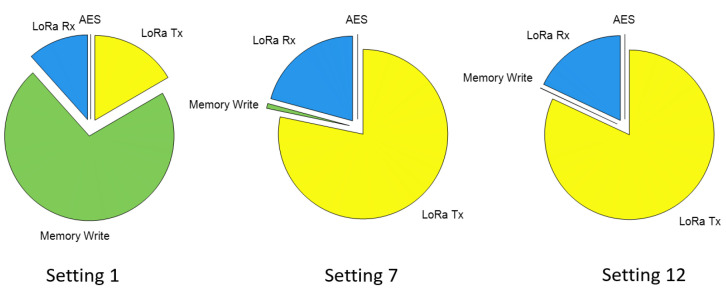
The energy consumption breakdown for the secure implementation.

These results show that it is not even possible to update a firmware image of this size with LoRa settings 11 and 12 on a single charge. The power consumption decreases dramatically with each setting however, motivating the need for ADR. The power consumption for the insecure implementation is similar to the secure implementation, which is justified by the relative energy breakdown shown in Figure 9. The energy consumption of the AES operation is very small because it is executed efficiently in hardware. Despite the LoRa receive time being much longer for the higher settings, it is less significant because the transmit current is 10x the receive current. Therefore, most of the energy expenditure comes from the packet acknowledgment operation under the high LoRa settings. As the settings decrease toward setting 1, the memory write operation begins to dominate because the RF communications become very small in duration.

### 3.3. Implementation

**Figure 10:**
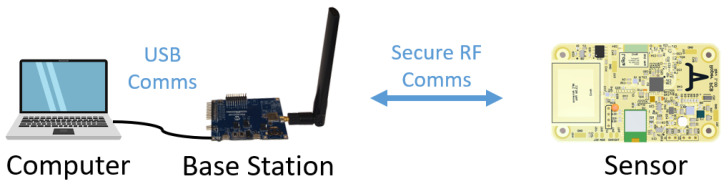
The experimental setup used to validate the firmware update process.

**Figure 11:**
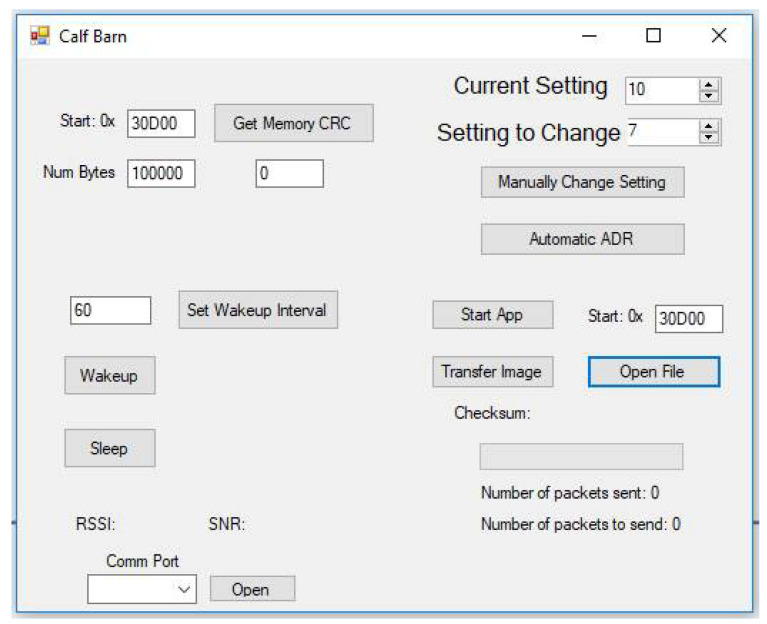
The PC interface to control the firmware update.

The secure firmware transfer process was validated in hardware using the test setup shown in Figure 10, which consists of a host computer, a Microchip SAMR34 Xplained evaluation board, and a Roper sensor printed circuit board (PCB) powered by a 200 mAh lithium polymer battery. The computer controls the update process via a .NET program which initiates the exchange, transmits the images, and then requests execution of the new application code. The host computer user interface that is used to test the prototype is shown in Figure 11. In order to securely transfer the update, the host program parses the new firmware update file, encrypts the firmware image, generates the MAC, and then transfers the encrypted image via Universal Serial Bus (USB) to the base station. The SAMR34 Xplained Pro evaluation board serves as the base station and acts as a USB-to-LoRa converter to handle RF communications at 915 MHz. The last byte of the USB message indicates the transmit and receive setting, and then all other bytes are transmitted over LoRa. In addition, the device data received by the base station is relayed asynchronously to the host along with the measured RSSI and signal-to-noise ratio (SNR) data. This custom code was developed and then loaded onto the base station.

The firmware for the cattle monitoring device represents 30% of the firmware image while the boot loader represents 25% of the image. Therefore, the constraints discussed earlier allow the next firmware update to be stored in internal flash memory. The sensor operation executes after the ADR is complete and the initial code load address has been initialized. An interrupt is generated when a packet is received, which allows the sensor to validate the packet, decrypt the data, and then write it to flash. Upon receiving a valid packet, an acknowledgement is sent to the base station to confirm that a successful transfer occurred. After the complete image has been transferred, a CRC is computed on the new code image. If the calculated CRC matches the transmitted CRC, a start application command is sent, which causes the sensor to reset and begin executing the new application code.

**Figure 12:**
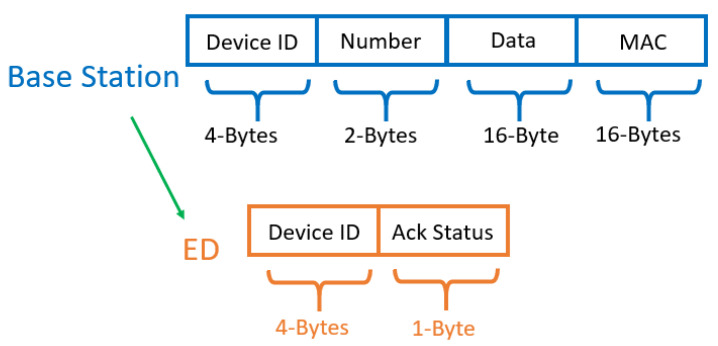
This shows the packet structures for exchanging the firmware image.

In order to validate the modeling, the energy consumption is measured for the firmware update process using settings 7 through 11. The results are shown in Table 4. The total energy consumption is estimated by measuring the battery open circuit voltage (OCV) before and after the firmware update. We then used a third-order OCV-to-battery-capacity mapping function to approximate the change in capacity. The firmware image used in the update is 106.6 kB which requires 6666 packet transfers when using the packet format specified in Figure 12. The total number of packets is calculated by dividing 106.6kB by 16 since 16-bytes of firmware data are transferred per packet. The hardware results are within 13% of those obtained from simulations except for setting 7. The most notable sources of error are given as follows: (1) The hardware experienced packet re-transmissions after failed transfers that were not present in the simulations. (2) The hardware experienced additional delay with the receiver on but without data being received. The root cause of this issue is attributed to delays in the processing time between the PC program and the base station. (3) The OCV-to-capacity model is temperature dependent, but we only applied the mapping under room temperature conditions. Any deviation in temperature would shift this capacity measurement slightly.

## 4. Discussion

### 4.1. Security Assessment

A malicious actor could try to steal the embedded firmware to access data or the proprietary embedded algorithms stored on the device, or they might attempt to load malicious firmware. The firmware, encrypted with AES by a herd specific key $K_{h}$, is exposed when it is broadcast over the air using LoRa by the base station, and when it resides on the device. Therefore, the attacker would only be able to collect the encrypted firmware image if they are able to track the ADR process to determine the base-station-to-device communication settings. An alternative attack is to spoof the base station by impersonating a device in need of a firmware update, and then collecting the encrypted firmware image. However, the protocol requires the initial exchange of messages to be signed with a device-specific key $K_{c}$; therefore, this attack would fail without the key because the base station would refuse to send the firmware update.

When the device receives the firmware image, it stores it in external memory. Storing an encrypted image consumes additional power since it must be decrypted to verify the message integrity. Moreover, the stored image is decrypted again before committing it to flash. An alternative is to decrypt the image during reception, and then store it unencrypted. The drawback here is that this enables a malicious actor to access the unencrypted image if they obtain access to the device. Note that the dual decryption operations are not required if the firmware update is stored in internal flash, but this is only possible if the firmware image meets the criteria from Equation (4). In the event that a device from the herd is stolen, it would be prudent to update $K_{h}$ for the entire herd in case the device is compromised using invasive techniques.

The second major security concern is loading malicious firmware onto the device. The malicious firmware could be as simple as code that disables the device causing a permanent DoS attack, or the malicious code could broadcast the wrong location so the system is unaware that cattle are being stolen. As a countermeasure, a MAC is appended to messages from the base station during the initial exchange which prevents unauthorized attempts to upload firmware to the device, unless $K_{c}$ becomes compromised. In the event that a malicious actor waits until after the initialization occurs to begin attempting to transfer a malicious image, the transfer would be unsuccessful because it would also need to be signed using $K_{c}$. Note that using $K_{c}$ to sign the update prevents multiple devices from being updated at the same time since $K_{c}$ is unique to each cattle. The code transfer cannot be signed with $K_{h}$ because this leaves the system open to replay attacks where the adversary could load old firmware versions onto the device without knowing $K_{h}$. A summary of this analysis is provided in Table 5.

### 4.2. Conclusions and Future Work

A secure and reliable firmware update methodology is proposed for mobile, energy-constrained LoRa devices. The secure code transfer and execution steps were simulated and then implemented on a battery-powered LoRa device. The most critical aspect of ensuring energy efficient updates is using ADR to find the optimal RF setting with the highest available data rate. Energy consumption scales non-linearly with the settings, making it worthwhile to invest time in converging to the optimal RF setting. The proposed secure authentication and encryption features add only an incremental burden on total energy consumption. The sensor AES computations also consume a negligible amount of time and energy. The most significant security burden is associated with the increased reception time of the MAC address. The proposed methodology could be improved by reducing the number of acknowledgements sent by the LoRa device since transmitting these impose the greatest energy burden. Fewer acknowledgements improves performance in the nominal scenario but introduces risk and redundancy if packets are frequently dropped or have transmission errors. Future work will integrate ADR during the data exchange as a means of maintaining the optimal RF settings when the LoRa device is mobile.

## Figures and Tables

**Table 1:** The time required to update a 128 kB firmware image.

Time to Transfer Firmware for a 128 kB Image				
**LoRa SF**	**6**	**8**	**10**	**12**
Time	16 min	46 min	2.49 h	8.36 h
**FSK (bps)**	**19,200**	**57,600**	**115,200**	**300,000**
Time	53.3 s	17.7 s	8.88 s	3.41 s

**Table 2:** The settings and timeout values used for adaptive data rate (ADR).

Setting Number	Modulation Type	Setting Configuration	Master Timeout	Device Timeout
0	FSK	300 kbps	0.1 s	0.4 s
1	FSK	200 kbps	0.1 s	0.4 s
2	FSK	115.2 kbps	0.1 s	0.4 s
3	FSK	57.6 kbps	0.1 s	0.4 s
4	FSK	19.2 kbps	0.1 s	0.4 s
5	FSK	9.6 kbps	0.1 s	0.4 s
6	LoRa	SF = 6	0.1 s	0.4 s
7	LoRa	SF = 7	0.2 s	0.4 s
8	LoRa	SF = 8	0.25 s	0.8 s
9	LoRa	SF = 9	0.4 s	1.6 s
10	LoRa	SF = 10	0.7 s	2.8 s
11	LoRa	SF = 11	1 s	4 s
12	LoRa	SF = 12	3 s	12 s

**Table 3:** The settings used for modeling the firmware update parameters.

Function	Time	Average Current
AES Encode	200 μs	6.5 mA
AES Decode	196 μs	6.5 mA
Flash Read	170 μs	6.5 mA
Flash Write	3.735 ms	25.5 mA
LoRa Transmit	varies	100 mA
LoRa Receive	varies	10 mA

**Table 4:** The energy consumed doing a firmware update at each Long Range (LoRa) setting.

Setting	Start OCV	Stop OCV	Measured Energy	Expected Energy
7	4.19 V	4.09 V	266.63 J	200.2 J
8	4.09 V	92.3 V	430.7 J	389 J
9	4.18 V	3.96 V	709.06 J	684.5 J
10	4.18 V	3.82 V	1166.14 J	1350 J
11	4.17 V	3.29 V	2669.2 J	2664 J

**Table 5:** The summary of the security risks and mitigation.

Objective	Method	Mitigation
Get Firmware	Observe firmware update by capturing it over RF.	The update is encrypted so it is meaningless to the observer after it is collected.
Get Firmware	Falsely claim to be a cattle that needs a firmware update.	The attacker would not have so it would fail the initial authentication.
Get Firmware	Read the firmware from the external memory chip.	The image is still encrypted external to the chip so it would not reveal any useful information.
Change Firmware	Falsely claim to be the base station and update the firmware on a sensor.	The malicious actor could not compose a valid MAC to pass the initial exchange.
Change Firmware	Wait until after the initial exchange and begin adding new firmware to the device.	Each data packet is signed using $K_{c}$ so no un-authenticated packets would ever be accepted.

## Data Availability

The data used to generate the plots and figures can be accessed by contacting the author at heegerds@unm.edu.
